# Commercial Laboratory Reproducibility of Serum CTX in Clinical Practice

**DOI:** 10.1002/jbm4.10225

**Published:** 2019-08-28

**Authors:** Sahar M Hindi, Eric Vittinghoff, Anne L Schafer, Stuart Silverman, Douglas C Bauer

**Affiliations:** ^1^ Division of Endocrinology Medical Subspecialties Institute, Cleveland Clinic Abu Dhabi Abu Dhabi United Arab Emirates; ^2^ Department of Epidemiology and Biostatistics University of California, San Francisco San Francisco CA USA; ^3^ Medical Service Endocrinology and Metabolism Section, San Francisco Veterans Affairs Health Care System San Francisco CA USA; ^4^ Department of Medicine, Division of Endocrinology and Metabolism University of California, San Francisco San Francisco CA USA; ^5^ Cedars Sinai Medical Center UCLA School of Medicine Los Angeles CA USA; ^6^ Department of Medicine, Division of General Internal Medicine University of California, San Francisco San Francisco CA USA

**Keywords:** BIOCHEMICAL MARKERS OF BONE TURNOVER; CTX; LABORATORY REPRODUCIBILITY

## Abstract

In 2011, the International Osteoporosis Foundation and the International Federation of Clinical Chemistry and Laboratory Medicine selected serum collagen type‐I crosslinked C‐peptide (s‐CTX) as the reference standard for bone resorption. This study aimed to determine the within and between laboratory reproducibility for s‐CTX assays. To create standardized pools, serum was collected from 10 premenopausal and 10 postmenopausal women. Premenopausal sera were pooled to approximate a population with normal bone turnover; postmenopausal sera were pooled to approximate a population with high bone turnover; and a third pool was created from an equal proportion of the pre‐ and postmenopausal pools. Multiple identical aliquots from each pool were created and frozen; all were labeled as routine clinical specimens. To evaluate longitudinal laboratory reproducibility, an identical aliquot from each of the three pools was sent to four US commercial laboratories on five dates over a 6‐month period. To evaluate within‐run reproducibility, each lab received five identical aliquots from each pool on the fifth date. Three labs (Mayo, ARUP, and Quest) used the Roche Diagnostics Elecsys assay, and one (Esoterix/LabCorp) used the IDS‐iSYS assay. Reproducibility was assessed using the coefficient of variation (CV) with 95% confidence intervals (CIs). Labs were unaware of the investigation. Across labs, mean s‐CTX values were 423, 533, and 480 pg/mL for the premenopausal, postmenopausal, and mixed pools, respectively, but the means differed between labs (*p* < 0.001). The premenopausal pool longitudinal CVs ranged from 5.0% to 14.9%; the postmenopausal pool CVs ranged from 3.4% to 19.3%; and the mixed pool CVs ranged from 3.3% to 16.0%. The longitudinal reproducibility for Esoterix/LabCorp was higher (CV 13.9%; 95% CI, 10.1% to 22.2%) than for the other labs. Within‐run CVs were also higher for Esoterix/LabCorp (CV 8.6%; 95% CI, 6.3% to 13.6%) compared with the other labs (CVs 2.1% to 6.2%). In conclusion, the reproducibility of s‐CTX varied across US commercial labs, and was poorer for Esoterix/LabCorp, which used the IDS assay, compared with the other three labs, which used the Roche assay. © 2019 The Authors. *JBMR Plus* published by Wiley Periodicals, Inc. on behalf of American Society for Bone and Mineral Research.

## Introduction

The field of bone turnover markers (BTMs) has developed considerably over the past decade. A significant amount of research has suggested that BTMs may provide useful information on fracture risk and may also have the potential to predict response to treatment for osteoporosis.^1^ These findings have secured a place for the use of BTMs in clinical research and research trials of new therapies as secondary endpoints of treatment efficacy. However, the use of BTMs in clinical practice has been limited by a number of factors, including the heterogeneity of assays and laboratory quality control. This variability profoundly limits the application of research findings to individualized patient care.^2^, ^3^, ^4^, ^5^, ^6^

To address some of the uncertainties regarding the clinical utility of BTMs, the International Osteoporosis Foundation (IOF) and the International Federation of Clinical Chemistry and Laboratory Medicine (IFCC) have recommended that reference markers, measured by standardized assays, be adopted for use in clinical trials and observational studies in osteoporosis to (1) enhance laboratory consistency, (2) broaden the international experience of the clinical application of BTMs to osteoporosis, and (3) facilitate their inclusion in routine clinical practice. In 2011, the IOF–IFCC selected serum collagen type‐I cross‐linked C‐peptide (s‐CTX) as the reference standard for bone resorption and serum amino‐terminal type‐I procollagen (s‐PINP) as the reference standard for bone formation.^2^

An important next step beyond the identification of reference BTMs is the standardization of the measurement of each marker with the aim of obtaining comparable values over time for each marker irrespective of the laboratory in which the measurement is made or the method utilized.^2^, ^7^ Over the last decade, many of the traditional BTM immunoassays have been automated, improving technical performance and increasing their availability. Nevertheless, analytical aspects, such as within‐ and between‐batch precision, accuracy, and standardization, remain problematic.^8^ Potential contributors to these variations include, but are not limited to, the specific analytical methods (eg, automated versus nonautomated platform), sample collection (eg, sample handling procedures), and laboratory performance (eg, sample analysis, calibration).^5^, ^6^, ^7^, ^8^

Previously, we reported substantial differences across US commercial labs when assessing the reproducibility of urine NTx and serum bone‐specific alkaline phosphatase.^9^ In the current study, we aimed to extend our previous findings to determine the within‐ and between‐laboratory variability of measurements of s‐CTX performed by US commercial labs under routine clinical conditions.

## Materials and Methods

### Participants

Pre‐ and postmenopausal women (postmenopausal women at least 10 years from last menstrual period), who were generally in good health, were recruited via advertising flyers posted around a large academic medical center. Volunteers were excluded if they were using pharmacotherapy for osteoporosis, defined as the current use of estrogen, calcitonin, a selective estrogen receptor modulator (SERM), bisphosphonates, denosumab, or teriparatide. The use of calcium and/or vitamin D supplements, as well as contraceptives, was permitted. Participants were also excluded if they had self‐reported major medical conditions known to affect bone turnover, specifically Paget disease, chronic kidney disease, end‐stage renal disease, hyperthyroidism, hyperparathyroidism, and active inflammatory disease. Individuals with a recent fracture were not identified or excluded from enrollment. All volunteers provided written informed consent after reading an information sheet that described the minimal risks involved in participation. The Institutional Review Board of the University of California, San Francisco (UCSF) approved the study protocol prior to initiation of the study.

### Creation of serum pools

To minimize the interfering effects of medications or other factors specific to a single volunteer, sera from individuals were pooled. Three pools of sera were created from specimens obtained from 20 volunteers (10 premenopausal women and 10 postmenopausal women) to approximate populations with normal and high levels of bone turnover. A third pool was created by equally mixing sera from pre‐ and postmenopausal women to approximate a population with intermediate levels of bone turnover.

To create the pools of sera, fasting blood was collected before 10 a.m. in six red‐top serum separator tubes from each participating woman. The blood was then allowed to clot at room temperature for 15 to 20 min, then was immediately placed on ice, centrifuged for 15 min, and separated. The serum from each individual participant was stored at –80°C in 10‐mL tubes until all volunteer samples were collected and ready for pooling. Once all volunteer samples were collected, the sera from each participant was thawed and pooled on the same day. Using a clean disposable pipette, sera from the 10 premenopausal women were pooled into a sterile flask and stirred for 10 min in an ice‐water bath. This process was repeated with sera from the 10 postmenopausal volunteers using a separate pipette and flask. To create the mixed pool of sera, an equal volume of serum from the premenopause pool and the postmenopause pool was drawn and combined into a third flask, then mixed 40 times with a glass stirrer. The resulting three pools of sera (premenopause, postmenopause, and mixed) were then divided into 0.5‐mL aliquots and placed into cryovials, flash‐frozen, and stored at –80°C. Each cryovial was labeled in a manner mimicking clinical send‐outs, including source‐masked research ID numbers, sample type (serum), and a fictitious collection date to coincide with the date of shipment to each lab.

### Selection of commercial laboratories

Four US laboratories were selected for investigation, each a recognized high‐volume commercial laboratory (ie, performs the bone marker assay at least 2 days per week and successfully participates in a proficiency‐testing program for the bone marker assay being investigated) that offers s‐CTX testing using an automated platform: ARUP Laboratories (Salt Lake City, UT, USA); Esoterix Laboratory Services (Calabasas Hills, CA, USA), which was acquired by Laboratory Corporation of America (LabCorp, Burlington, NC, USA); Mayo Medical Laboratories (Rochester, MN, USA); and Quest Diagnostics (Nichols Institute, San Juan Capistrano, CA, USA). To prevent bias and observer variability, the laboratories were unaware of the investigation; source‐masked identifiers were used for all specimens; and the specimens were sent to labs via a third‐party outpatient private clinical practice as routine clinical specimens ordered by outpatient providers would be sent. To control for analytical variability, specimens were mixed, processed, and stored following established standardized procedures. The laboratories were not aware of the study objectives and were paid in full via the standard contractual arrangements in place with the third‐party clinical practice.

### Timing of specimen delivery

Each laboratory was sent one archived aliquot of serum from each pool (ie, three uniquely labeled specimens) on five dates over a 6‐month period to assess longitudinal (between‐run) variability of the marker measurements. To minimize the differences in sample thawing, samples were sent to all labs on a day of the week chosen so that all samples would be analyzed on the same day. Specimens were sent every six to seven weeks. For all laboratories, on the fifth and final date, five uniquely labeled identical serum specimens from each pool (for a total of 15 specimens) were sent to each laboratory to assess within‐run variability of the marker measurement.

### s‐CTX assay

Each of the four labs used one of two US Food and Drug Administration‐ (FDA‐) approved assays run on an automated platform for s‐CTX measurements using an electro‐chemiluminescence immunoassay (ECLIA). ECLIAs use two specific monoclonal antibodies directed against the amino acid sequence of EKAHD‐β‐GGR, where the aspartic acid residue (D) is β‐isomerized. To obtain a specific signal in the ECLIA, two changes of EKAHD‐β‐GGR must be crosslinked.

Three of the four laboratories used the Beta‐CrossLaps assay manufactured by Roche Diagnostics (Indianapolis, IN, USA), whereas one laboratory (Esoterix/Labcorp) used the Serum CrossLaps assay manufactured by Immunodiagnostic Systems Holdings PLC (IDS; Tyne and Wear, UK). Because Esoterix has been acquired by LabCorp, any clinical specimens sent to LabCorp are forwarded to Esoterix for analysis (therefore, assays sent to either lab are run on the same assay and are thus referred to simply as Esoterix/LabCorp).

The laboratories communicated the results by fax to the third‐party outpatient clinic, as would be done for routine clinical specimens. These results were then forwarded to the principal investigator for unmasked tabulation and analysis. S‐CTX values were reported by all labs in whole numbers and in picograms per milliliter (pg/mL).

### Statistical analysis

Means, SDs, and coefficients of variation (CVs, defined as SD/mean) with 95% confidence intervals (CIs)^10^ were calculated using R Version 3.3.2 (R Foundation for Statistical Computing, Vienna, Austria). In estimating within‐lab CVs using all three pools, as well as overall CVs, SDs were calculated with respect to the within‐pool means. We used asymptotic tests to assess heterogeneity of the CVs between pools and labs,^11^ and simulation to obtain prediction intervals for the calculated reduction in marker values for the range of observed CVs. In post hoc sensitivity analyses, we omitted results for Esoterix/LabCorp in comparing longitudinal heterogeneity of the CVs across labs, and omitted an outlying result for the postmenopausal pool obtained by Quest in estimating longitudinal within‐lab CVs. Finally, we used a linear mixed model to assess between‐lab heterogeneity in average measurements after accounting for variability because of pool. This analysis was implemented in Stata Version 15.1 (StataCorp LLC, College Station, TX, USA).

## Results

### Reference range results

We requested that each lab provide the reference range for its s‐CTX assay. For postmenopausal women, the reported reference ranges (all in pg/mL) were 142 to 1351 for Esoterix/Labcorp and 104 to 1008 for ARUP and Mayo. Quest did not provide postmenopausal normative data. For premenopausal women, Esoterix/Labcorp and Mayo reported reference ranges of 112 to 738 and 25 to 573, respectively, and the premenopausal reference range for ARUP and Quest was 60 to 650 for women ages 18 to 39 years and 40 to 465 for women ages 40 to 49 years.

### Longitudinal reproducibility

Longitudinal reproducibility was evaluated by sending one specimen from each pool to each of four labs on five separate dates (only the first of the five identical specimens sent on the fifth send‐out was included in the longitudinal analysis). CVs for the premenopausal pool varied from 5.0% to 14.9%, whereas those from the mixed and postmenopausal pools varied from 3.0% to 16.0% and from 3.4% to 19.3%, respectively. There was statistically significant between‐pool heterogeneity of the CVs for Quest only (*p* = 0.007; Table 1).

**Table 1:** Longitudinal Within‐Lab Reproducibility of s‐CTX at Four US Commercial Labs^a^

Lab	Pool	Assay	*N*	Mean ± SD (pg/mL)	CV, % (95% CI)	Heterogeneity of CV between pools^b^
ARUP	PRE	ECLIA, Roche	5	394 ± 19.8	5.0 (3.0 to 14.6)	*p* = 0.47
ARUP	MIX	ECLIA, Roche	5	449 ± 13.7	3.0 (1.8 to 8.8)	
ARUP	POST	ECLIA, Roche	5	496 ± 16.9	3.4 (2.0 to 9.8)	
Esoterix/LabCorp	PRE	ECLIA, IDS	5	518 ± 77.0	14.9 (8.9 to 44.8)	*p* = 0.94
Esoterix/LabCorp	MIX	ECLIA, IDS	5	573 ± 91.4	16.0 (9.5 to 44.8)	
Esoterix/LabCorp	POST	ECLIA, IDS	5	645 ± 87.2	13.5 (8.1 to 40.4)	
Mayo	PRE	ECLIA, Roche	5	430 ± 29.8	6.9 (4.1 to 20.1)	*p* = 0.38
Mayo	MIX	ECLIA, Roche	5	486 ± 18.5	3.8 (2.3 to 11.0)	
Mayo	POST	ECLIA, Roche	5	540 ± 25.7	4.8 (2.9 to 13.7)	
Quest	PRE	ECLIA, Roche	5	408 ± 35.0	8.6 (5.1 to 25.0)	*p* < 0.001
Quest	MIX	ECLIA, Roche	5	448 ± 19.6	4.4 (2.6 to 12.6)	
Quest	POST	ECLIA, Roche	5	451 ± 86.3	19.3 (11.5 to 60.5)^c^	

s‐CTX = Serum collagen type‐I crosslinked C‐peptide; CV = coefficient of variation; PRE = premenopausal pool; POST = postmenopausal pool; MIX = equal proportion of the pre‐ and postmenopausal pools; ECLIA = electro‐chemiluminescence immunoassay.

a Calculated from five identical serum specimens sent to each lab five times over a 6‐month period.

b Lab‐specific *p* value for heterogeneity of CVs for premenopausal, mixed, and postmenopausal pools.

c When a single outlier is removed from the postmenopausal pool, Quest mean ± SD is 500 ± 24.2 and CV (95% CI) is 4.8 (2.7 to 18.2).

For mixed and postmenopausal pools, there was significant heterogeneity of longitudinal CVs between the four labs (Table 2). When results from Esoterix/LabCorp, which used a different assay, was removed from the calculation, the heterogeneity of CVs between labs was no longer statistically significant for the premenopausal (*p* = 0.52) or mixed (*p* = 0.73) pools, but remained significant for the postmenopausal pool (*p* < 0.001) (Table 2, third column).

**Table 2:** Longitudinal Between‐Lab Reproducibility of s‐CTX Within Each Pool

Pool	*p* value for heterogeneity of CVs (all 4 labs)^a^	*p* value for heterogeneity of CVs (after removal of Esoterix/LabCorp)^a^
PRE	0.08	0.52
MIX	<0.001	0.73
POST	0.001	<0.001

s‐CTX = Serum collagen type‐I crosslinked C‐peptide; CV = coefficient of variation; PRE = premenopausal pool; POST = postmenopausal pool; MIX = equal proportion of the pre‐ and postmenopausal pools.

a *p* value for heterogeneity of longitudinal CVs between labs.

**Figure 1:**
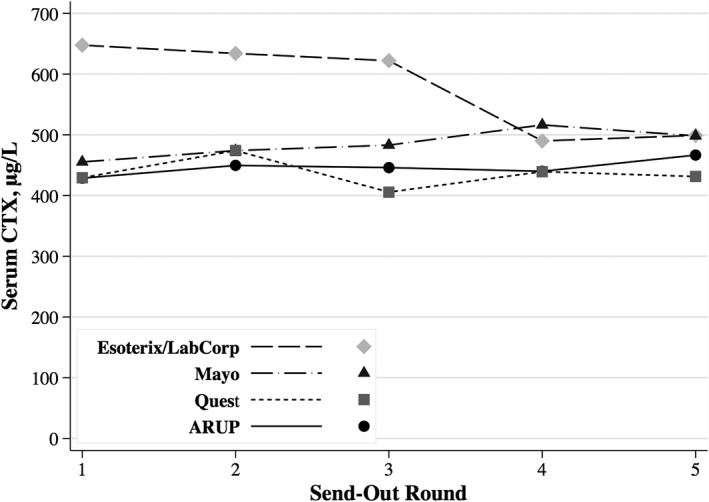
Six‐month longitudinal reproducibility of serum collagen type‐I crosslinked C‐peptide (s‐CTX) at four US commercial labs (all pools combined).

Combining the premenopausal, mixed, and postmenopausal pool results for each laboratory (Fig. 1), the overall longitudinal within‐lab CVs for s‐CTX were recalculated (Table 3) and ranged from 3.5% (ARUP) to 13.9% (Esoterix/Labcorp). The *p* value for overall heterogeneity of the CVs across labs was <0.001, both before and after excluding Esoterix/LabCorp. In addition, the *p* value for overall heterogeneity of the combined means for all three pools across labs, which ranged from 436 (Quest) to 579 (Esoterix/LabCorp) was <0.001 (Table 3).

**Table 3:** Longitudinal Within‐Lab Reproducibility of s‐CTX (All Pools Combined)

Lab	*N*	Mean ± SD	Heterogeneity of means between all 4 labs	Heterogeneity of means after removal of Esoterix/Lab Corp	CV, % (95% CI)	Heterogeneity of CV between all 4 labs	Heterogeneity of CV between labs after removal of Esoterix/Lab Corp
ARUP	15	446 ± 15.8	*p* < 0.001	*p* < .001	3.5 (2.6 to 5.6)	*p* < 0.001	*p* < 0.001
Esoterix/LabCorp	15	579 ± 80.5			13.9 (10.1 to 22.2)		
Mayo	15	485 ± 23.9			4.9 (3.6 to 7.8)		
Quest	15	436 ± 53.7			12.3 (9.0 to 19.6)		

s‐CTX = Serum collagen type‐I crosslinked C‐peptide; CV = coefficient of variation.

### Within‐run reproducibility

Cross‐sectional within‐run reproducibility for each lab was evaluated by sending five identical specimens, each with unique patient identifiers, from each of three pools on one date (chosen as the fifth and final send‐out date). For the premenopausal pool (Table 4), cross‐sectional within‐run CVs varied from 1.1% (Mayo) to 10.4% (Esoterix/LabCorp). For the mixed pool, CVs varied from 1.3% (ARUP) to 9.3% (Esoterix/LabCorp). For the postmenopausal pool, CVs varied from 2.8% (ARUP) to 8.0% (Esoterix/LabCorp). There was statistically significant heterogeneity of CVs among pools at Mayo only (Table 4), which appeared to have a higher CV (6.8%) for the mixed pool compared with the pre‐ and postmenopausal pools (*p* = 0.002).

**Table 4:** Cross‐Sectional Within‐Run Reproducibility of s‐CTX at Four US Commercial Labs^a^

Lab	Group	Assay	*N*	Mean ± SD (pg/mL)	CV, % (95% CI)	Heterogeneity of CV between pools^b^
ARUP	PRE	ECLIA, Roche	5	412 ± 7.0	1.7 (1.0 to 4.9)	*p* = 0.21
ARUP	MIX	ECLIA, Roche	5	459 ± 5.9	1.3 (0.8 to 3.7)	
ARUP	POST	ECLIA, Roche	5	515 ± 14.3	2.8 (1.7 to 8.0)	
Esoterix/LabCorp	PRE	ECLIA, IDS	5	411 ± 42.9	10.4 (6.2 to 30.7)	*p* = 0.84
Esoterix/LabCorp	MIX	ECLIA, IDS	5	514 ± 48.0	9.3 (5.6 to 27.3)	
Esoterix/LabCorp	POST	ECLIA, IDS	5	574 ± 46.0	8.0 (4.8 to 23.3)	
Mayo	PRE	ECLIA, Roche	5	450 ± 5.1	1.1 (0.7 to 3.3)	*p* = 0.002
Mayo	MIX	ECLIA, Roche	5	479 ± 32.8	6.8 (4.1 to 19.9)	
Mayo	POST	ECLIA, Roche	5	543 ± 17.1	3.1 (1.9 to 9.1)	
Quest	PRE	ECLIA, Roche	5	373 ± 24.6	6.6 (3.9 to 19.1)	*p* = 0.51
Quest	MIX	ECLIA, Roche	5	421 ± 21.9	5.2 (3.1 to 15.0)	
Quest	POST	ECLIA, Roche	5	487 ± 18.8	3.9 (2.3 to 11.1)	

s‐CTX = Serum collagen type‐I crosslinked C‐peptide; CV = coefficient of variation; PRE = premenopausal pool; POST = postmenopausal pool; MIX = equal proportion of the pre‐ and postmenopausal pools; ECLIA = electro‐chemiluminescence immunoassay.

a Calculated from five identical serum specimens set to each lab on one day.

b Lab‐specific *p* value for heterogeneity of CVs for premenopausal, mixed, and postmenopausal pools.

There was also statistically significant heterogeneity of cross‐sectional within‐run CVs among labs within each pool (*p* < 0.05; Table 5). After Esoterix/LabCorp was removed from the calculation, significant variability within the premenopausal (*p* < 0.001) and mixed (*p* = 0.017) pools remained.

**Table 5:** Cross‐Sectional Within‐Run Reproducibility of s‐CTX Within Each Pool

Pool	*p*‐value for heterogeneity of CVs (all four labs)^a^	*p*‐value for heterogeneity of CVs (after removal of Esoterix/LabCorp)^a^
PRE	<0.001	<0.001
MIX	0.014	0.017
POST	0.031	0.75

s‐CTX = Serum collagen type‐I crosslinked C‐peptide; CV = coefficient of variation; PRE = premenopausal pool; POST = postmenopausal pool; MIX = equal proportion of the pre‐ and postmenopausal pools.

a *p*‐value for heterogeneity across labs for cross‐sectional within run CVs.

Combining results from all three pools sent to each lab on the fifth and final shipment, cross‐sectional within‐run CVs for s‐CTX were calculated (Table 6). CVs varied from 2.1% (ARUP) to 8.6% (Esoterix/LabCorp). The *p* value for heterogeneity across labs was <0.001 both before and after excluding Esoterix/LabCorp.

**Table 6:** Cross‐Sectional Within‐Lab Reproducibility for s‐CTX (All Pools Combined)

Lab	*N*	Mean ± SD	CV, % (95% CI)	Heterogeneity of CV between all 4 labs^a^	Heterogeneity of CV between labs after removal of Esoterix/Labcorp^a^
ARUP	15	462 ± 9.5	2.1 (1.5 to 3.3)	*p* < 0.001	*p* < 0.001
Esoterix/LabCorp	15	500 ± 42.8	8.6 (6.3 to 13.6)		
Mayo	15	491 ± 20.9	4.3 (3.1 to 6.7)		
Quest	15	427 ± 26.6	6.2 (4.6 to 9.8)		

s‐CTX = Serum collagen type‐I crosslinked C‐peptide; CV = coefficient of variation.

a *p* value for heterogeneity across labs for cross‐sectional within run CVs.

### Simulation of monitoring with s‐CTX during treatment

In the setting of treatment monitoring with s‐CTX, the following hypothetical example provides some insight into the clinical implications of our study. If s‐CTX measurements were obtained (using the same lab) prior to and 3 months after starting bisphosphonate treatment, we used the observed laboratory reproducibility in this study to simulate 95% prediction intervals for the observed percent reduction in s‐CTX when the true value falls by 50%. In other words, this analysis provides the plausible ranges of reported results for a true 50% reduction in s‐CTX. In this hypothetical example, the prediction interval for the percent reduction in s‐CTX estimated by a lab with a longitudinal CV of 3.5% (ARUP) is an estimated 45% to 55%. In contrast, for a laboratory with a longitudinal CV of 13.9% (such as Esoterix/LabCorp), the plausible range would be 35% to 71%.

## Discussion

In this masked study of identical specimens, both the longitudinal and cross‐sectional reproducibility of s‐CTX was highly variable at high‐volume US commercial labs. For each lab, variability was generally similar for premenopausal, postmenopausal, and mixed pools, but was significantly greater for the one laboratory utilizing the IDS assay (ie, Esoterix/LabCorp). Conversely, s‐CTX reproducibility was better among the three labs utilizing the Roche assay, but statistically significant differences between laboratories were documented for specific pools even among those using the same s‐CTX assay. We cannot determine if this variability is attributable to the assay used or to the laboratory performing the assay. However, our results confirm that in clinical practice, serial measurements utilizing the same assay and laboratory will result in more stable results over time.

A limitation of the present study is the small number of labs evaluated. However, the labs evaluated represent high‐volume, well‐recognized commercial labs collectively responsible for a significant proportion of s‐CTX assays conducted in the United States. Another limitation is that longitudinal reproducibility was tested over a period of 6 months, and not a longer period as might be anticipated in clinical practice. A third limitation of the present study is that although two BTMs are recommended by the IOF‐IFCC,^2^ we only assessed s‐CTX, recognizing that there are currently no automated assays for s‐PINP that are FDA‐approved. However, specimens have been archived for analysis at a later date once automated assays for s‐PINP receive FDA‐approval. Therefore, the laboratory reproducibility of s‐PINP remains an area to be studied. Finally, our study lacks a sample with a markedly elevated bone turnover level, as the pool created to reflect high turnover turned out to be relatively close in value to the pool with low turnover.

A strength of this study is the testing of multiple pooled samples for s‐CTX representing normal to elevated marker values to approximate populations encountered in routine clinical practice. Furthermore, the pooling of specimens minimized interfering effects of medications or other factors specific to a single volunteer. Although pooled samples do not behave exactly like individual patient samples, the aim of our study was to examine heterogeneity attributed to the laboratory and not to the individual patient. Pooling in this setting is therefore a useful mechanism to eliminate the confounding factor of individual patient heterogeneity.

Another strength of our study is that the commercial laboratories were blinded to the investigation. The laboratories were not aware of the study objectives and were paid in full via the standard contractual arrangements in place with the third‐party clinical practice. Thus, our protocol differs from announced and unblinded proficiency testing conducted according to the Clinical Laboratory Improvement Amendments and the College of American Pathologists, where reproducibility may be at its best. The purpose of blinding the commercial laboratories to the investigation in this, as well as our previous study,^9^ was to prevent bias and observer variability. Moreover, we wanted to ensure that the samples were processed exactly the same as routine clinical samples. Finally, it should be noted that CTX is more stable in EDTA plasma than in serum. For this reason, the National Bone Health Alliance (NBHA) prefers CTX in EDTA plasma if a lab is not able to process a sample immediately.^12^ However, we used serum CTX in our study to best reflect clinical practice in the United States because the majority of US commercial labs use serum for CTX processing. Moreover, in line with NBHA recommendations to reduce preanalytical variability, we took careful measures to ensure standardized sample handling and patient preparation.

BTMs are valuable tools for assessing the dynamic nature of bone. They may enhance the estimation of fracture risk when paired with static BMD data, and may independently provide valuable information about treatment response and efficacy when monitoring therapy in osteoporosis management.^1^, ^3^, ^4^, ^5^ Despite the clinical potential of BTMs, current assays for BTMs lack commonly accepted standardizations, limiting the application of BTMs to routine clinical practice.^6^, ^7^, ^8^, ^9^ A number of organizations, including the IOF and IFCC, have recognized the need to advance the field of BTMs and address the limitations of their use for routine clinical practice.^2^, ^7^ Poor reproducibility is a major barrier to the use of biochemical markers of bone turnover in clinical practice. This challenge in clinical practice is further complicated by the fact that information about the particular assay and platform used by a given lab may not be disclosed. For example, in this study only the results from ARUP and Mayo included the assay employed. Further, the results of laboratory proficiency testing are not widely available to practicing clinicians.

In conclusion, in this blinded analysis using identical aliquots of pooled serum, we found that the longitudinal and within‐run reproducibility of s‐CTX measurements differed across four US commercial laboratories, even after accounting for different assays. Based on these differences, we estimate that some labs may have suboptimal reproducibility, which reduces the utility of serial s‐CTX measurements. Therefore, in clinical practice, the importance of utilizing the same highly reproducible laboratory over time should be emphasized to optimize the utility of serial s‐CTX measurements obtained for the purpose of monitoring therapy. Before the routine use of s‐CTX can be recommended, additional studies are needed to determine the causes of laboratory variability.

## Disclosures

SS has received payments from Roche Diagnostics and Roche Pharma to speak at Roche symposia in the past. SS is currently Principal Investigator of a drug holiday study funded by the National Osteoporosis Foundation. All other authors state that they currently have no disclosures.
